# Strategies to minimize fall-related injuries in older adults at risk of falls: the Falling Safely Training study

**DOI:** 10.1093/geroni/igaf122.2641

**Published:** 2025-12-31

**Authors:** Jacob Sosnoff, Lingjun Chen, Shelley Bhattacharya, James Fang, Jianghua He, Abbas Tabatabaei, Neil Alexander, Tobia Zanotto

**Affiliations:** University of Kansas Medical Center, Kansas City, Kansas, United States; University of Kansas Medical Center, Kansas City, Kansas, United States; University of Kansas Medical Center, Kansas City, Kansas, United States; University of Kansas Medical Center, Kansas City, Kansas, United States; University of Kansas Medical Center, Kansas City, Kansas, United States; University of Kansas Medical Center, Kansas City, Kansas, United States; University of Michigan, Ann Arbor, Michigan, United States; University of Kansas Medical Center, Kansas City, Kansas, United States

## Abstract

The Falling Safely Training (FAST) program is designed to reduce fall-related injury in older adults at risk of injury. This study evaluated its feasibility and preliminary efficacy in mitigating fall-related injury. Twenty-four older adults (72.53 ± 5.39 years) were randomized to four weeks of FAST training, which focused on progressive safe-falling strategies, or to an active control group that completed balance training via the modified Otago program. Participants underwent experimentally induced falls in a laboratory setting at baseline, post-intervention, and three-month follow-up. Head and hip accelerations were captured via motion tracking, and head impact occurrences were assessed through video screening. Eleven of 12 FAST participants completed training without adverse events. Compared to the control group, the FAST group had a greater reduction in head impact risk (odds ratio = 0.10, 95% CI: 0.02, 0.61, p = 0.012) and lower head acceleration post-intervention (Δ = -9.54 m/s², p = 0.028). Hip acceleration decreased in both groups (p < 0.001). Group differences in demographic variables were assessed using Mann–Whitney U and Chi-square tests, while linear mixed models examined acceleration changes and mixed logistic regression evaluated head impact occurrence. These findings suggest that FAST is a feasible and effective intervention for reducing fall-related head impact risk in older adults. By targeting safe-falling strategies, this approach addresses a critical gap in fall prevention programs and offers a promising avenue for injury mitigation. Future research should explore its long-term cost-effectiveness and community implementation.

